# Disrupted Cervicovaginal Microbiota: Its Role in *Chlamydia trachomatis* Genital Infection and Associated Reproductive Outcomes

**DOI:** 10.3390/ijms262110635

**Published:** 2025-10-31

**Authors:** Rafaela Rodrigues, Ana Rita Silva, Carlos Sousa, Nuno Vale

**Affiliations:** 1 PerMed Research Group, RISE-Health, Faculty of Medicine, University of Porto, Alameda Professor Hernâni Monteiro, 4200-319 Porto, Portugal; rafaela24sofia@hotmail.com (R.R.); ana.rita.silva@unilabs.com (A.R.S.); carlos.sousa@unilabs.com (C.S.); 2 RISE-Health, Department of Community Medicine, Health Information and Decision (MEDCIDS), Faculty of Medicine, University of Porto, Rua Doutor Plácido da Costa, 4200-450 Porto, Portugal; 3 Molecular Diagnostics Laboratory, Unilabs Portugal, Centro Empresarial Lionesa Porto, Rua Lionesa, 4465-671 Leça do Balio, Portugal; 4 Laboratory of Personalized Medicine, Department of Community Medicine, Health Information and Decision (MEDCIDS), Faculty of Medicine, University of Porto, Rua Doutor Plácido da Costa, 4200-450 Porto, Portugal

**Keywords:** cervicovaginal microbiota, *chlamydia trachomatis* genital infections, sexually transmitted infections, host-microbe interactions, women reproductive health

## Abstract

*Chlamydia trachomatis* (CT) remains the most commonly reported bacterial sexually transmitted infection (STI) globally, with particularly high incidence among adolescents and young adults. In Europe, CT cases have continued to rise over the past decade, despite ongoing public health efforts in prevention and screening. Screening coverage, however, remains inconsistent across countries. CT infections are often asymptomatic, especially in women, yet can lead to serious CT-related reproductive complications if left untreated, including pelvic inflammatory disease (PID), tubal factor infertility, and ectopic pregnancy. Emerging evidence highlights the cervicovaginal microbiota as a key factor influencing susceptibility to STIs, including CT infection, its progression, and associated outcomes. A *Lactobacillus*-dominated microbiota, particularly *L. crispatus*, is well-known to be a protective factor against CT acquisition, whereas vaginal dysbiosis, characterized by a depletion of these species and an overgrowth of anaerobes, such as *Gardnerella vaginalis*, *Atopobium vaginae*, and *Prevotella* spp., has been linked to increased CT acquisition risk, reduced immune control, and impaired infection resolution. Interaction between microbial communities and host immunity may modulate whether CT infections spontaneously clear, persist, or progress into pathological conditions. This review explores the natural history of CT genital infection in women, emphasizing the role of cervicovaginal dysbiosis in disease progression and reproductive sequelae. By integrating current knowledge about resident cervicovaginal microbes, host-microbe interaction, and CT-related reproductive outcomes, we discuss how microbiota-targeted strategies, including probiotic or microbiome-modulating strategies, may complement current CT prevention, diagnosis, and treatment approaches.

## 1. Introduction

*Chlamydia trachomatis* (CT) infections are the most common sexually transmitted bacterial infections worldwide [1]. Recent epidemiological data indicate a continuous rise in CT prevalence across Europe, alongside other sexually transmitted infections (STIs), particularly, gonorrhea and syphilis [2]. According to the most recent European Centre for Disease Prevention and Control (ECDC) report regarding Annual Epidemiological Reports on STIs, between 2013 and 2023, there was a 13% increase in reported CT cases. Of note, men who have sex with men (MSM) accounted for approximately 20% of these cases, a trend that continues to raise public health concerns [3]. In contrast, data from the United States Centers for Disease Control and Prevention (CDC) reveal a different scenario. Between 2022 and 2023, a 7.2% decrease in *Neisseria gonorrhoeae* infections was observed, while CT case numbers remained relatively stable [4]. These differences may partly reflect variations in screening coverage, reporting practices, healthcare access, and population-level sexual health behaviors [5].

Chlamydial genital infections are often asymptomatic. It is estimated that 70–95% of infected women and over 50% of infected men do not exhibit symptoms, although some individuals may develop clinical manifestations [6]. In women, symptoms of CT genital infection may include abnormal vaginal discharge, pelvic pain, dysuria. In males, typical presentations include urethral discharge, dysuria, and discomfort or pain in the genital area [7]. Due to the asymptomatic nature and high transmissibility of CT infection, public health strategies focused on prevention, condom promotion, and sexual health education are crucial. Awareness campaigns remain among the most cost-effective tools to combat the rising prevalence of STIs [8]. In parallel with education, the implementation of routine screening programs is essential for the early detection and timely treatment of infections, especially in women, to avoid long-term reproductive complications, such as pelvic inflammatory disease (PID), ectopic pregnancy, and infertility [9]. These preventive strategies offer clinically beneficial as well as economic advantages, particularly in regions with limited healthcare resources and socioeconomic barriers that may delay access to care [10,11,12].

This review focuses on CT genital infections in reproductive-age women, where the most severe sequelae include PID, ectopic pregnancy, infertility, and even tumorigenesis [13,14,15]. Several behavioral and biological factors contribute to the risk of infection, including specific sexual practices and partner-related variables [16]. Importantly, increasing attention has recently been given to the role of the cervicovaginal microbiota in modulating susceptibility to STIs, immune responses of cervicovaginal tract and long-term outcomes of the genital infection [13,17]. Emerging evidence suggests that variations in vaginal microbial communities, particularly the dominance or absence of protective *Lactobacillus* species, may influence both the likelihood of contracting CT and the progression of the infection [18,19].

The present work aims to characterize the interaction between the cervicovaginal microbial ecosystem and the female reproductive health, with a particular focus on CT-related outcomes. Herein we explore how shifts in microbial composition and host immune response at the vaginal mucosa influence susceptibility to CT infection and the risk of long-term complications. Finally, we discuss current and emerging microbiome-targeted therapeutic strategies for chlamydial infections.

## 2. The Role of Cervicovaginal Microbiota in Female Reproductive Tract Health

The vaginal microbiota is a dynamic ecosystem, composed of numerous microorganisms and exhibits considerable interindividual variability over time [20,21]. Notably, the term microbiome refers to this community of microorganisms, along with their collective genomes and metabolic products, which coexist within the host vaginal environment, maintaining a functionally balanced ecosystem [22].

Cervicovaginal microbiota composition varies throughout a woman’s life, influenced by several physiological, hormonal, and environmental factors [23,24]. In healthy reproductive-age women, however, the vaginal microbiota is typically characterized by low microbial diversity, and it is colonized mainly by *Lactobacillus* species, particularly *L. crispatus*, *L. gasseri*, *L. jensenii*, and *L. iners* [25,26]. These bacteria play a vital role in maintaining an acidic vaginal pH, of approximately 3.5 to 4.5, primarily through the production of lactic acid, as well as other antimicrobial compounds and metabolites such as bacteriocins, organic acids, and hydrogen peroxide [27,28]. All these substances help preventing colonization by pathogenic bacterial, viral and parasitic agents, and contribute to immune regulation and mucosal barrier integrity [29,30]. In detail, the cervicovaginal epithelium surface is covered by mucus, which is enriched of mucins, antimicrobial proteins, and immunoglobulins, playing a key role in vaginal health as a physical and biochemical barrier, preventing infections and facilitating host-microbiota interactions [31]. In addition, by adhering to the vaginal epithelium, *Lactobacillus* spp. forms a physical barrier, competing for adhesion sites, preventing the attachment of harmful bacteria, and competing for nutrients as well, effectively starving potential pathogens, reducing their ability to prosper in cervicovaginal epithelium [32,33].

As previously mentioned, the composition of the vaginal microbiota is not static and is influenced by various factors, including age, hormonal status, ethnicity, geographic location, sexual behavior, pregnancy, antibiotic use, and diet [34,35]. For instance, estrogen, which is produced in higher amounts during the women’s reproductive years, promotes glycogen accumulation in the vaginal epithelium, creating a favorable environment for *Lactobacillus* proliferation [36]. The glycogen present in vaginal epithelial cells is catabolized by human enzymes (α-amylase), forming small molecules (maltose, maltotriose and α-dextrines) that are metabolized by *Lactobacillus* species to lactic acid, reducing vaginal pH [29,37]. Indeed, this negative correlation between glycogen and vaginal pH has been demonstrated analyzing women’s vaginal fluid, as demonstrated by Mirmonsef and colleagues [38]. In contrast, during menopause, estrogen levels decline, leading to reduced glycogen availability, subsequently, a decrease in *Lactobacillus* abundance, ultimately leading to an increase in microbial diversity and vaginal pH [39]. Antibiotic use can also reduce *Lactobacillus* populations, thereby disrupting microbial homeostasis [40,41,42,43]. These changes collectively contribute to an increased susceptibility to sexually transmitted infections, particularly to *Chlamydia trachomatis*, which will be a focus of discussion in the present manuscript [36,44].

Given the high degree of variability in microbial composition across individuals, their life stages, and owing to advances in molecular biology and DNA sequencing technologies, researchers have developed standardized classification frameworks to better describe and compare vaginal microbiota profiles, also known as vaginotypes or cervicotypes [45,46]. One widely adopted system is the Community State Types (CSTs) classification, which organizes vaginal microbiota into distinct categories based on dominant bacterial species, determined by 16S rRNA gene sequencing (Table 1) [46]. CST-I is typically dominated by *L. crispatus*, CST-II by *L. gasseri*, CST-III by *L. iners*, and CST-V by *L. jensenii* [20]. In contrast, CST-IV is characterized by low or absent *Lactobacillus* species, and higher diversity of facultative and/or obligate anaerobes such as *Gardnerella vaginalis*, *Atopobium vaginae*, and *Prevotella*, a pattern often linked to a condition known as vaginal dysbiosis, which will be further explored herein [45,47]. Notably, both CST-III and CST-IV comprise subgroups with distinct microbiological and clinical features, some of which have been increasingly linked to adverse reproductive outcomes and pathological conditions, namely bacterial vaginosis, as further detailed by Dong and colleagues [35].

Importantly, to support the reproducibility of CST classification across studies, the VALENCIA (VAginaL community state typE Nearest CentroId clAssifier) tool was introduced. It uses an algorithm trained on a large reference dataset to robustly classify cervicovaginal microbiota profiles into these different CSTs. This has become a valuable tool in vaginal microbiome research, providing a consistent framework for investigating associations between microbial composition, infection susceptibility, and reproductive health outcomes [49].

### 2.1. Vaginal Dysbiosis

**Figure 1:**
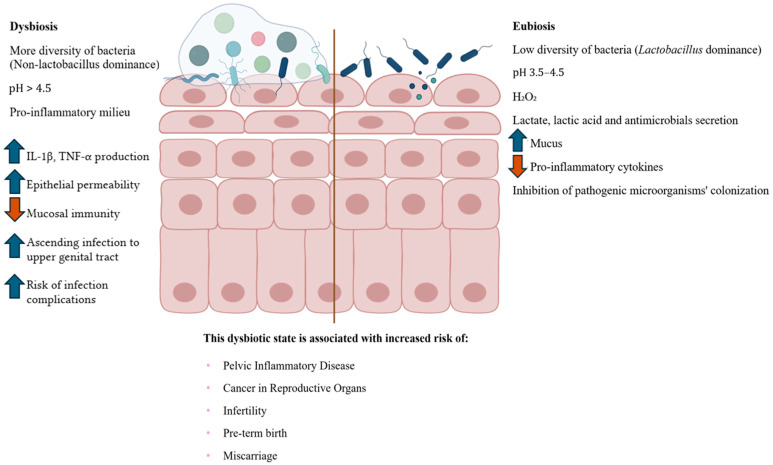
Cervicovaginal dysbiosis and eubiosis. The right side illustrates a healthy cervicovaginal epithelium, characterized by *Lactobacillus* spp. dominance, which helps maintain an acidic pH, produce protective metabolites (e.g., lactic acid and hydrogen peroxide), increase mucus production, reinforce epithelial barrier integrity, and modulate immune homeostasis. On the left, dysbiosis is depicted, marked by the depletion of *Lactobacillus* spp. and overgrowth of anaerobic bacteria, such as *Gardnerella*, *Prevotella*, and *Atopobium*. This microbial imbalance leads to elevated pH, epithelial barrier disruption, and increased expression of pro-inflammatory cytokines (e.g., IL-1β, TNF-α), r resulting in mucosal inflammation and microbial ascension to the upper genital tract. These mechanisms contribute to increased susceptibility to STIs, such as CT infection and its complications, including pelvic inflammatory disease (PID), miscarriage, preterm birth, infertility, and gynecological cancers [50,56]. The central vertical line indicates the conceptual boundary between dysbiotic and eubiotic states. Figure created using BioRender—https://www.biorender.com/ (accessed on 30 June 2025).

While a *Lactobacillus*-dominated microbiota is associated with vaginal health, its disruption can lead to a dysbiosis state, which significantly alters the local immune microenvironment and increases susceptibility to pathogens, including *Chlamydia trachomatis* (Figure 1). Thus, vaginal dysbiosis is characterized by a decrease in *Lactobacillus* dominance and an overgrowth of anaerobic bacteria such as *Prevotella* spp., *Gardnerella vaginalis*, *Mycoplasma hominis*, *Peptostreptococcus* spp., *Fusobacterium* spp., *Porphyromonas* spp., and *Mobiluncus* spp. [50]. These bacteria produce some specific molecules, including sialidases, proteases, and short-chain fatty acids, which degrades the mucosal and cervical epithelial barriers, and induces a pro-inflammatory microenvironment, triggering pro-inflammatory cytokine secretion and immune cells differentiation into pro-inflammatory profile [51]. Importantly, dysbiosis is associated with clinical conditions such as bacterial vaginosis, aerobic vaginitis, candidiasis, and increased susceptibility to STIs, including chlamydial infections [52,53]. Furthermore, dysbiosis has significant implications for reproductive health. It has been linked to pregnancy complications, including preterm birth, and can contribute to infertility and poor gynecological outcomes [3,50,54,55].

### 2.2. Interplay Between Vaginal Microbiota, Host Immunometabolism, and CT Infection

Understanding the mutualistic relationship between the host and its vaginal microbiota is essential to identify the mechanisms that maintain health or drive disease [20]. The host provides a favorable niche for microbial colonization, while the microbiota plays a crucial role in maintaining women’s health, particularly through immune modulation, epithelial protection, and pathogen exclusion [57]. Within this microenvironment, *Lactobacillus* contributes to homeostasis by preserving cervical epithelial barrier integrity and local immune response regulation, as previously discussed. Importantly, the vaginal mucosa is not merely a physical barrier, but also a functionally active immune tissue, composed of various resident immune cells [58]. In a healthy state, these immune cells remain in a state of controlled homeostasis [59]. Conversely, cervicovaginal dysbiosis disrupts this balance, compromises mucosal defenses, and triggers inflammatory responses, thereby increasing susceptibility to opportunistic pathogens, as already mentioned [60,61].

**Figure 2:**
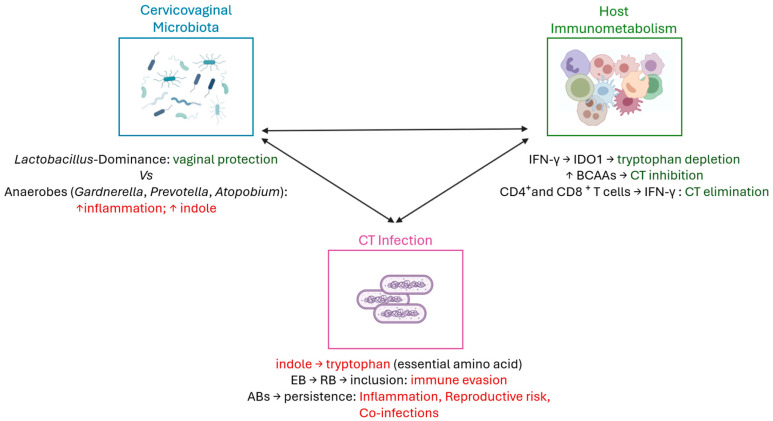
Interplay between vaginal microbiota, host immunometabolism, and *Chlamydia trachomatis* infection. CD4+ helper T-cells and CD8+ cytotoxic T-cells are responsible for CT clearance [67,68,69,70]. Additionally, a *Lactobacillus*-dominated vaginal microbiota contributes to mucosal homeostasis by maintaining epithelial integrity, modulating local immune responses, and limiting pathogen colonization [50]. In contrast, anaerobic bacteria can increase local inflammation and indole production, creating a favorable environment for CT infection [60,61]. Indole could be used by CT to synthesize tryptophan, an essential amino acid for its growth [71,72,73,74]. Also, thanks to its biphasic cell cycle, the conversion of infectious elementary bodies (EBs) into replicative reticulate bodies (RBs) within the inclusion enables immune evasion [14,63]. Under unfavorable conditions, CT can convert into aberrant bodies (ABs), leading to persistent infection, chronic inflammation, reproductive complications, and increased risk of co-infections [71]. Host immunometabolic factors, including IFN-γ–mediated tryptophan depletion and branched-chain amino acid (BCAA) signaling, interact with microbial metabolites to influence CT infection outcomes [75,76,77,78,79,80]. Figure created using BioRender—https://www.biorender.com/ (accessed on 20 October 2025).

*Chlamydia trachomatis* is an obligate intracellular bacterium with a unique biphasic developmental cycle, alternating between the infectious elementary body (EB) and the replicative reticulate body (RB) [62]. Throughout most of its life cycle, the bacterium resides within a specialized membrane-bound compartment known as an inclusion, which protects it from the host immune surveillance and facilitates immune evasion, thereby contributing to persistent infections, as described in previous studies [14,63]. This developmental strategy is key to understanding CT capacity to establish chronic infections. A central point is to understand why some women are able to naturally clear CT infection, while others develop persistence and the infection progress to serious reproductive sequelae [64]. The ability to spontaneously clear CT likely depends on a combination of host’s immune responses, metabolic mechanisms occurring in the genital tract, and the host’s cervicovaginal microbiota (Figure 2) [65,66].

#### 2.2.1. CT Infection Clearance

The natural clearance of CT infection could be mediated by the host’s innate and adaptative immune responses [67]. Upon infection, epithelial cells and the resident immune cells recognize CT via pattern recognition receptors (PRRs), such as Toll-like receptor 9 (TLR9), triggering pro-inflammatory cytokine signaling cascades [68]. This pro-inflammatory microenvironment is essential to recruit neutrophils, macrophages, dendritic cells, and natural killer (NK) cells, which together activates adaptive immune response, particularly, CD4+ helper T-cells and CD8+ cytotoxic T-cells [67]. Of note, these lymphocytes are responsible for bacterial clearance, particularly through the production of interferon-gamma (IFN-γ), a key cytokine in CT control [69,70].

One of the well-characterized mechanisms for controlling CT infection involves IFN-γ–induced depletion of tryptophan, mediated by the host enzyme indoleamine 2,3-dioxygenase 1 (IDO1) [65]. Upon IFN-γ stimulation, IDO1 catabolizes tryptophan into kynurenine, reducing amino acid availability in the intracellular environment. As CT is auxotrophic for tryptophan, this depletion stops bacterial replication and drives the formation of aberrant bodies (ABs), a persistent and non-infectious form of the bacterium [71]. However, certain urogenital CT strains retain a functional tryptophan synthase that can convert indole, which is produced by specific members of the vaginal microbiota, back into tryptophan [72]. This ability allows the bacteria to evade IFN-γ–mediated starvation, reinitiating its developmental cycle, and potentially reemerge from persistence [71,72]. Additionally, Wood and colleagues demonstrated, in vitro, that direct inhibition of tryptophanyl-tRNA and leucyl-tRNA synthetases is sufficient to induce CT persistence, even in the absence of IFN-γ [73]. This highlights CT’s ability to sense amino acids deprivation and enter a reversible and a non-replicative state (ABs). More recently, Jordan and colleagues provided in vivo evidence for this mechanism by showing that women who naturally cleared the infection had lower cervicovaginal tryptophan levels, despite having low IFN-γ expression [74]. This piece of evidence suggests that host-driven metabolic pressures may contribute to bacterial clearance. Furthermore, several studies report that low levels of branched-chain amino acids (BCAAs), such as leucine and isoleucine, are associated with CT growth inhibition and the induction of persistence in culture models [75,76]. Recent studies highlight the intricate balance among BCAAs as a critical factor in CT metabolism and persistence. Banerjee and colleagues identified a transporter (CTL0225) belonging to an ancient family of bacterial amino acid transporters, as pivotal for the uptake of leucine, isoleucine, and valine. Intriguingly, their work shows that excess leucine or isoleucine inhibits CT growth, but this inhibition can be reversed by the addition of valine, suggesting competitive interactions at the transporter level. Altogether, this reinforces the concept that BCAAs homeostasis not only supports bacterial growth but also influences the pathogen’s ability to enter a persistent state, further linking host metabolic environment to infection outcomes [77]. Of note, BCAAs (leucine, isoleucine, and valine), are essential amino acids involved in distinct pathways: protein synthesis, energy metabolism, and in the regulation of immune and inflammatory responses [78]. Beyond the structural role, BCAAs act as key metabolic signals that influence the cellular antimicrobial defense, acting on cellular immunity and cytokine production [79]. Alterations in BCAAs metabolism have been reported during both bacterial and viral infections, suggesting a role in host susceptibility modulation and pathogen persistence. In line with this, variations in BCAAs availability may influence the outcome of CT infections, potentially tipping the balance between bacterial clearance and chronic infection [80].

While most studies have focused on the mechanisms underlying CT persistence, the biological mechanisms responsible for natural clearance remain less understood [66]. However, as previously discussed, emerging evidence suggests that a robust mucosal immune response, characterized by IFN-γ production, effective CD4^+^ T cell activation, and a *Lactobacillus*-dominated microbiota, may promote pathogen elimination. *Lactobacillus* species play a protective role in the vaginal epithelium, not only by reduction local inflammation, but also by producing lactic acid and bacteriocins, which help maintain epithelial integrity and reduce cellular susceptibility to pathogens [81,82,83,84,85]. This mechanism of spontaneous clearance of CT may be interconnected with tryptophan metabolism, as a *Lactobacillus*-dominated microbiota produces less indole, potentially limiting CT’s ability to synthesize tryptophan and thereby restricting its survival [53,86]. Concomitantly, the absence of indole-producing anaerobes further reduces bacteria ability to synthesize tryptophan during IFN-γ-mediated starvation, tipping the balance toward bacterial clearance [87]. Moreover, metabolic profiles enriched in antimicrobial amino acid signaling, including controlled levels of BCAAs, may contribute to infection control by modulating immune responses and directly affecting bacterial persistence [88]. Taken together, these insights support the notion that successful clearance is not merely an immunological event, but the result of a tightly regulated immunometabolism and microbial interplay within the cervicovaginal niche (Figure 2).

#### 2.2.2. Cervicovaginal Dysbiosis, Infection Risks and Reproductive Outcomes

Cervicovaginal dysbiosis not only increases susceptibility to CT infection but also promotes a persistent pro-inflammatory environment that facilitates bacterial ascension and chronic pathology [89]. The depletion of protective *Lactobacillus* species, particularly *L. crispatus*, and overrepresentation of anaerobes such as *Gardnerella*, *Prevotella*, and *Atopobium*, contribute to local immune dysregulation [90]. These microbial shifts are associated with increased production of inflammatory mediators, such as interleukin-1β (IL-1β) and tumor necrosis factor-alpha (TNF-α), disrupting epithelial barrier integrity and amplifying mucosal permeability [60]. This altered environment facilitates CT entry and persistence, while impairing mucosal healing and adaptive immune resolution (Figure 1) [91].

Emerging evidence also links cervicovaginal microbial composition with adverse pregnancy outcomes [92,93,94]. Some studies have demonstrated that women with CST-IV or polymicrobial dysbiosis are at elevated risk of preterm birth, ectopic pregnancy, and other obstetric complications [50,95]. These outcomes appear to be mediated by chronic subclinical inflammation, local cytokine imbalances, and immune activation at the maternal–fetal interface [96].

Moreover, cervicovaginal dysbiosis has been associated not only with persistent CT infections and adverse reproductive outcomes, but also with increased susceptibility to co-infections, such as human papillomavirus (HPV), and a higher risk of developing cervical intraepithelial neoplasia [97,98]. Bacterial vaginosis, often characterized by CST-IV microbiota, may further amplify this risk by promoting chronic inflammation, mucosal disruption, and viral persistence [99]. Interestingly, emerging evidence suggests that *Lactobacillus* species may exert a protective effect beyond certain diseases. In detail, Xu and colleagues have demonstrated that *Lactobacillus* spp. are protector factors against ovarian cancer, potentially through modulation of local immune responses and maintenance of epithelial integrity [100]. In addition, microbiota imbalance has been proved as increasing the risk of ovarian malignancy [101]. Indeed, the influence of microbiota in gynecological cancers, including cervical and ovarian tumors, has been increasingly explored in the recent literature [102,103].

Altogether, these findings underscore the importance of vaginal microbial balance not only in infection control, but also in reproductive and obstetric health.

## 3. Therapeutic Strategies and Future Directions

Although current treatment of CT infection relies primarily on antibiotic therapy, typically with azithromycin or doxycycline, these approaches do not address the underlying factors contributing to reinfection or persistence, such as cervicovaginal dysbiosis [7]. In fact, growing evidence supports the idea that the cervicovaginal microbiota is not only a key regulator of women’s reproductive health but also a promising therapeutic target in the context of STIs, such as CT infection [18,104,105]. Dysbiosis, in particular the depletion of *Lactobacillus crispatus* and overgrowth of anaerobic bacteria, is increasingly viewed as a modifiable factor that contributes to CT persistence and adverse clinical outcomes [106]. Accordingly, restoring a healthy microbial community has emerged as a strategic focus in disease prevention and management (Table 2) [107,108,109].

In this context, microbiota-based interventions are increasingly recognized not only as therapeutic tools to reverse dysbiosis, but also as preventative strategies to maintain a *Lactobacillus*-dominant vaginal environment and reduce susceptibility to genital infections (Table 2) [110,111]. Among the most explored approaches are probiotic and prebiotic therapies aimed at enhancing *Lactobacillus*-dominated communities, reducing inflammation in the cervicovaginal epithelium [109,110,112]. Some studies have evaluated the administration of specific *Lactobacillus* strains, such as *L*. *crispatus* CTV-05, showing favorable effects on microbiota restoration, epithelial barrier integrity, and reduced susceptibility to STIs, including CT [113,114]. Current evidence highlights that probiotic interventions are most effective when administered after completion of antibiotic therapy, allowing recolonization of the vaginal niche once pathogenic bacteria have been eradicated [125].

Notably, vaginal microbiota homeostasis appears to be influenced by the gut microbiota [107]. This interconnection suggests that targeting the gut microbial ecosystem may also represent an indirect but effective therapeutic approach to restore vaginal eubiosis [115,116]. Importantly, cervicovaginal dysbiosis is now understood to be a reversible condition in many cases, and targeted microbial therapies may facilitate a shift towards a more eubiotic state [117]. However, there are many challenges in this field, including limited colonization efficacy, strain-specific variability, and a lack of long-term follow-up data on clinical outcomes. In addition, therapeutic success of microbiota-based therapies may be influenced by multiple host-related factors, as the host’s hormonal status, sexual behavior, and immune profile [118,119].

In parallel, the field is also advancing toward precision medicine approaches, where microbiome-based risk stratification informs tailored interventions. Advances in cervicovaginal microbiome sequencing and computational modeling enable the development of personalized microbial risk scores, allowing for the stratification of women based on their susceptibility to CT persistence, reinfection, or complications [17,89]. These tools pave the way for future targeted interventions, including customized probiotic formulations and other microbiota-based therapies.

Beyond probiotics, vaginal microbiota transplantation (VMT) has recently emerged as an innovative strategy, particularly in women with recurrent or refractory dysbiosis [107,120]. VMT involves the transfer of cervicovaginal fluid from healthy donors to restore microbial homeostasis to recipients lacking a protective and healthy cervicovaginal flora [107]. Using an in vivo model, Chen and colleagues demonstrated the feasibility and efficacy of VMT or probiotic combination in restoring beneficial microorganisms and resolving dysbiosis-associated symptoms in animals with this condition [121]. Another promising strategy involves exploring the interaction between the microbiota and mucosal vaccine responses [122]. The composition of the local microbiota may influence both innate and adaptive immunity at mucosal surfaces, ultimately impacting vaccine efficacy [123]. Although conducted in the context of chlamydial ocular infection, the study by Inic-Kanada et al. demonstrated that *Lactobacillus rhamnosus* can act as a probiotic adjuvant, shaping the magnitude and quality of vaccine-induced immune responses. These findings support the broader concept that microbiota modulation may serve as a co-adjuvant strategy to improve vaccine efficacy, potentially applicable to genital *Chlamydia trachomatis* infections as well [124].

## 4. Conclusions

Current research increasingly supports the pivotal role of the cervicovaginal microbiota and host immunometabolism environment in shaping the outcomes of chlamydial genital infections. A *Lactobacillus*-dominated cervicovaginal flora, particularly enriched in *L. crispatus*, supports epithelial barrier integrity, limits inflammation, and favors spontaneous pathogens clearance. In contrast, dysbiotic profiles characterized by anaerobic overgrowth are associated with persistent and chronic infections, immune evasion, pro-inflammatory microenvironment and increased risk of long-term reproductive complications. Metabolic pathways, especially those involving tryptophan and branched-chain amino acids, further modulate the interplay between host and pathogen, influencing bacterial survival and immune evasion. These insights underscore the importance of viewing CT genital infection not only as a pathogen-driven process, but as one shaped by a dynamic and multifactorial host–microbe–metabolite communication. Although further studies are needed to validate these pathways in vivo and translate them into clinical strategies. From a therapeutic perspective, microbiota-targeted interventions hold promise because they may enhance natural immunity, improve vaccine efficacy, reduce CT-associated morbidity, and even reduce the incidence of other STIs. However, the strategies including VMT and vaccination approaches modulated by host microbial profiles, remain experimental and require robust clinical trials to confirm their efficacy and safety. Indeed, the most promising short-term interventions are those aiming to prevent or reverse dysbiosis through safe, strain-specific probiotics, potentially combined with conventional antibiotics. In the long term, precision medicine approaches, including personalized microbial risk profiles and immunometabolism modulation, may update the way to diagnose, prevent, and treat CT infections.

Therefore, CT genital infections should no longer be viewed solely as a pathogen-driven disease, but as a host-microbe-metabolite interplay, which means that target this communication offers a new frontier for more effective and personalized care in women’s reproductive health.

## Figures and Tables

**Table 1:** Cervicovaginal microbiota classification based on Community State Types (CSTs) and associated features [35,46,48].

Community State Type (CST)	Dominant Bacteria	Microbiological/Clinical Features
CST-I	*Lactobacillus crispatus*	Low diversity; stable community; associated with vaginal health and low inflammation.
CST-II	*Lactobacillus gasseri*	Moderate stability; protective but less so than CST-I.
CST-III	*Lactobacillus iners*	Transitional state; associated with both health and dysbiosis; can persist in inflammatory settings.
CST-IV	Diverse anaerobes (*Gardnerella*, *Atopobium*, *Prevotella*, etc.)	High diversity; low/no *Lactobacillus*; associated with bacterial vaginosis and increased infection risk.
CST-V	*Lactobacillus jensenii*	Less common; associated with vaginal health.

**Table 2:** Summary of current and emerging therapeutic strategies targeting cervicovaginal dysbiosis and *Chlamydia trachomatis* infection [7,107,108,109,110,111,112,113,114,115,116,117,118,119,120,121,122,123,124].

Therapeutic Strategy	Examples/Agents	Mechanism/Target	Current Limitations/Challenges
Antibiotic therapy	Azithromycin; Doxycycline	Eradication of *C. trachomatis*	Does not restore healthy microbiota; reinfection risk.
Probiotic therapy	*L. crispatus* CTV-05	Restoration of *Lactobacillus*-dominant microbiota; modulation of local immunity	Variable colonization success; strain-specific effects.
Prebiotic therapy	Oligosaccharides	Promote growth of beneficial *Lactobacillus* spp.	Limited human data.
Gut–vaginal axis modulation	Oral probiotics; Fecal Microbiota Transplantation	Indirect restoration of vaginal eubiosis through gut microbiota	Mechanisms not fully understood; Need for more fundamental studies.
Microbiota-informed precision medicine	Predictive microbial risk scores	Risk stratification based on cervicovaginal microbiome profile	Requires clinical validation.
Vaginal microbiota transplantation	Donor cervicovaginal fluid	Reintroduction of healthy cervicovaginal flora	Concerns regarding safety, donor screening, and ethical to be used in humans.
Microbiota–vaccine interaction	*L. rhamnosus* as mucosal adjuvant	Use of probiotics as immune adjuvants	Translational gap to clinical use.

## Data Availability

Not applicable.
